# Experimental Investigation of Gypsum Composites Incorporating Polymeric Rubber Waste from the Vulcanization Industry

**DOI:** 10.3390/polym18212648

**Published:** 2026-10-26

**Authors:** Karol Prałat, Justyna Ciemnicka, Michał Ciarkowski

**Affiliations:** Faculty of Civil Engineering, Mechanics and Petrochemistry, Warsaw University of Technology, Łukasiewicza 17, 09-400 Płock, Poland; justyna.ciemnicka@pw.edu.pl (J.C.); michal.ciarkowski.stud@pw.edu.pl (M.C.)

**Keywords:** gypsum composites, rubber waste, vulcanization industry, bending strength, compressive strength

## Abstract

The aim of this study was to comprehensively evaluate the influence of rubber waste originating from the vulcanisation industry on the mechanical performance of gypsum composites. Three rubber fractions (0–0.6 mm, 0.3–1.5 mm and 1–4 mm) were incorporated at contents ranging from 0% to 50% of binder mass. Increasing rubber content resulted in a systematic reduction in composite density, decreasing from 1534 kg/m^3^ for the reference material to 1204 kg/m^3^, 1128 kg/m^3^ and 1031 kg/m^3^ at 50% addition for the three fractions, respectively. Flexural strength dropped from 0.296 MPa to 0.059 MPa (fine fraction) and 0.033 MPa (coarse fraction), while compressive strength decreased from 4.556 MPa to 1.542 MPa and 1.370 MPa at 25% rubber content. These results demonstrate that both particle size and quantitative share of rubber significantly affect the mechanical response of the composite. Generalised regression-based correlations were developed for flexural and compressive strength as a function of density and rubber content, providing practical descriptive models for the analysed range. The findings offer a quantitative basis for further research on the incorporation of rubber waste into gypsum-based materials and for assessing their potential use in non-structural applications.

## 1. Introduction

Gypsum-based materials have for many decades constituted an important component of construction technologies, finding application both in finishing elements and in the prefabrication of lightweight structural components. Their popularity stems from favourable physical and functional properties, such as low weight, good workability, and short setting time. Research on the microstructure and mechanical properties of gypsum materials indicates that their behaviour largely depends on porosity, the type of binder, and hydration conditions [1,2]. Gypsum is also characterised by low energy demand during production, which makes it an environmentally friendly material compared with Portland cement. Despite these advantages, its use is limited by relatively low mechanical strength, brittle fracture behaviour, and low resistance to dynamic loading [3,4]. For this reason, intensive efforts are being made to identify methods of modifying gypsum that would improve its mechanical performance and broaden its range of applications.

One of the research directions that has gained particular attention in recent years is the use of waste materials as modifying additives. This approach aligns with the principles of the circular economy and sustainable development strategies, which promote the reuse of resources and the reduction in waste sent to landfills. Among waste materials, rubber originating from the vulcanisation industry is of particular interest; it is obtained mainly from the recycling of automotive tyres, post-production residues, and worn technical components. Rubber waste poses a significant environmental challenge due to its durability, resistance to degradation, and the large volume generated by the automotive industry [5,6]. The recycling of rubber and its reuse in mineral composites is therefore a solution that is both environmentally beneficial and technically justified.

Research on cement mortars, in which rubber constitutes one of the most frequently analysed additives, has been developing particularly intensively in recent years [7]. These studies include, among others, assessments of the influence of particle size, the degree of substitution, and the optimal dosage of the additive [8], as well as the analyses of the behaviour of structural elements with different geometries [9].

One of the most frequently studied applications is the partial replacement of conventional aggregates with recycled rubber in concrete mixtures [10,11,12]. In the case of gypsum materials, however, this issue appears less frequently, although it has also been attracting increasing interest [13]. The available research indicates that the introduction of rubber leads to a reduction in the bulk density of the composite, resulting in lighter materials [14,15]. Rubber in the form of granules or powder is characterised by elasticity [16], abrasion resistance, vibration-damping capability [17], and high elastic deformability [18].

The introduction of rubber into gypsum leads to the formation of a composite with modified mechanical and microstructural properties. The addition of rubber typically results in a reduction in the bulk density of the composite, which stems from the lower density of rubber compared with the gypsum binder. A decrease in compressive strength is also observed, which is typical for composites containing lightweight and elastic additives [19,20]. However, the authors of [20] demonstrate that the incorporation of carbon fibres improves the compressive strength of gypsum.

Rubber is a natural or synthetic polymer whose fundamental structural unit is isoprene, i.e., 2-methyl-1,3-butadiene. In natural rubber, isoprene units are linked into long chains in the cis-1,4 configuration, which imparts exceptional elasticity and the ability to undergo reversible deformations [21]. This raw material is obtained primarily from the *Hevea brasiliensis* tree, in which the polymer constitutes approximately 30–40% of the mass, and its properties arise from the high mobility of polymer chains, which in the unvulcanised state are soft and viscoelastic [22]. To obtain rubber with high strength, the material is subjected to vulcanisation, that is, the cross-linking of chains with sulfur, during which sulfur bridges form between adjacent polymer chains. The cross-link density determines the hardness, elasticity, and abrasion resistance of the final rubber; therefore, mixtures of natural and synthetic rubber are used in automotive tyres to achieve an optimal balance between flexibility and durability [23]. The most commonly used synthetic rubber is Styrene Butadiene Rubber (SBR), which exhibits good abrasion resistance and thermal stability, making it a primary component of tyre treads [24]. Another important polymer is Butadiene Rubber (BR), which enhances elasticity at low temperatures [25]. In the inner layers of tyres, Isobutylene Isoprene Rubber (IIR), commonly known as butyl rubber, is used due to its low gas permeability, ensuring airtightness [26]. Rubber mixtures also contain carbon black, which reinforces the polymer structure [27]. The chemical structures of the main rubbers used in tyre production are summarised in Table 1.

The mechanical properties of rubber depend on the length of the polymer chains and the degree of cross-linking, as an insufficient number of sulfur bridges results in excessive softness, whereas an excessive number leads to material brittleness. In tyre recycling, mechanical shredding is most commonly used, producing rubber granulate of various fractions that retains the structure of cross-linked rubber; consequently, it does not melt and cannot be reprocessed like a conventional thermoplastic. This cross-linked structure makes recycled rubber thermally and chemically stable, and the addition of rubber to construction materials such as gypsum can improve their crack resistance and increase their deformation capacity. Rubber particles act as an elastic dispersed phase that absorbs deformation energy and limits microcracking in gypsum. It should be noted, however, that the present study does not include direct measurements of strain, ductility, impact resistance or other parameters describing the deformation capacity of the composite. Therefore, any potential influence of rubber on deformation behaviour can only be considered qualitatively and should not be interpreted as a confirmed improvement. A detailed assessment of deformation resistance will require dedicated mechanical testing in the future. The properties of the composite may depend on the granulate size and its amount in the gypsum matrix. The chemical inertness of rubber ensures that it does not react with the components of gypsum, which contributes to the stability of the composite.

In the context of gypsum composites, it is particularly important to understand the relationship between the rubber content and the mechanical properties of the material. In the field of construction materials research, increasing attention is being devoted to the use of rubber waste as an additive modifying the properties of mineral binders. Within this research direction, numerous studies focus on incorporating rubber granulate or powder derived from tyre recycling into traditional binders used in construction. The introduction of rubber into gypsum also affects the microstructure of the composite. Transition zones with distinct porosity and adhesion characteristics are formed, which may lead to local stress concentrations. At the same time, the presence of rubber can limit the development of microcracks, thereby improving resistance to dynamic and impact loading.

The use of rubber originating from the vulcanisation industry as an additive to gypsum materials represents a promising research direction, combining environmental considerations with the potential to improve selected mechanical properties. A key aspect is to determine the influence of rubber content on compressive strength and flexural strength. The results of such studies may contribute to the development of new, more sustainable construction materials with an expanded range of applications.

Previous studies on the use of rubber waste have focused primarily on cement-based composites, whereas the literature concerning gypsum-based materials is limited and does not include analyses that simultaneously consider a broad range of rubber particle sizes and high replacement levels. Existing publications also do not provide generalised relationships enabling the estimation of the mechanical properties of gypsum based on density or rubber content. This study addresses this gap by presenting comprehensive tests of three rubber granulate fractions within the range of 5–50% of the binder mass and by proposing linear models that allow rapid prediction of composite strength without the need for destructive testing.

## 2. Materials and Methods

The main materials used in this work were: commercially available gypsum powder (90–99 wt.% of bassanite, hemihydrate CaSO_4_·0.5H_2_O), with properties consistent with the safety data sheet [28], and ground rubber originating from the vulcanisation industry in three different fractions [29,30,31].

### 2.1. Gypsum

The material applied during the research was building gypsum (Dolina Nidy, Pinczów, Poland) precisely described in [32,33]. This gypsum is used in construction, renovation, and completion works inside buildings. It is also used to produce mortars and prefabricated elements. Grey-yellow gypsum powder, widely available on the market and meeting the standard requirements, was selected. Some physical properties of the gypsum powder used in the research can be found in [34] and in the safety data sheet [28].

### 2.2. Ground Rubber

**Figure 1:**
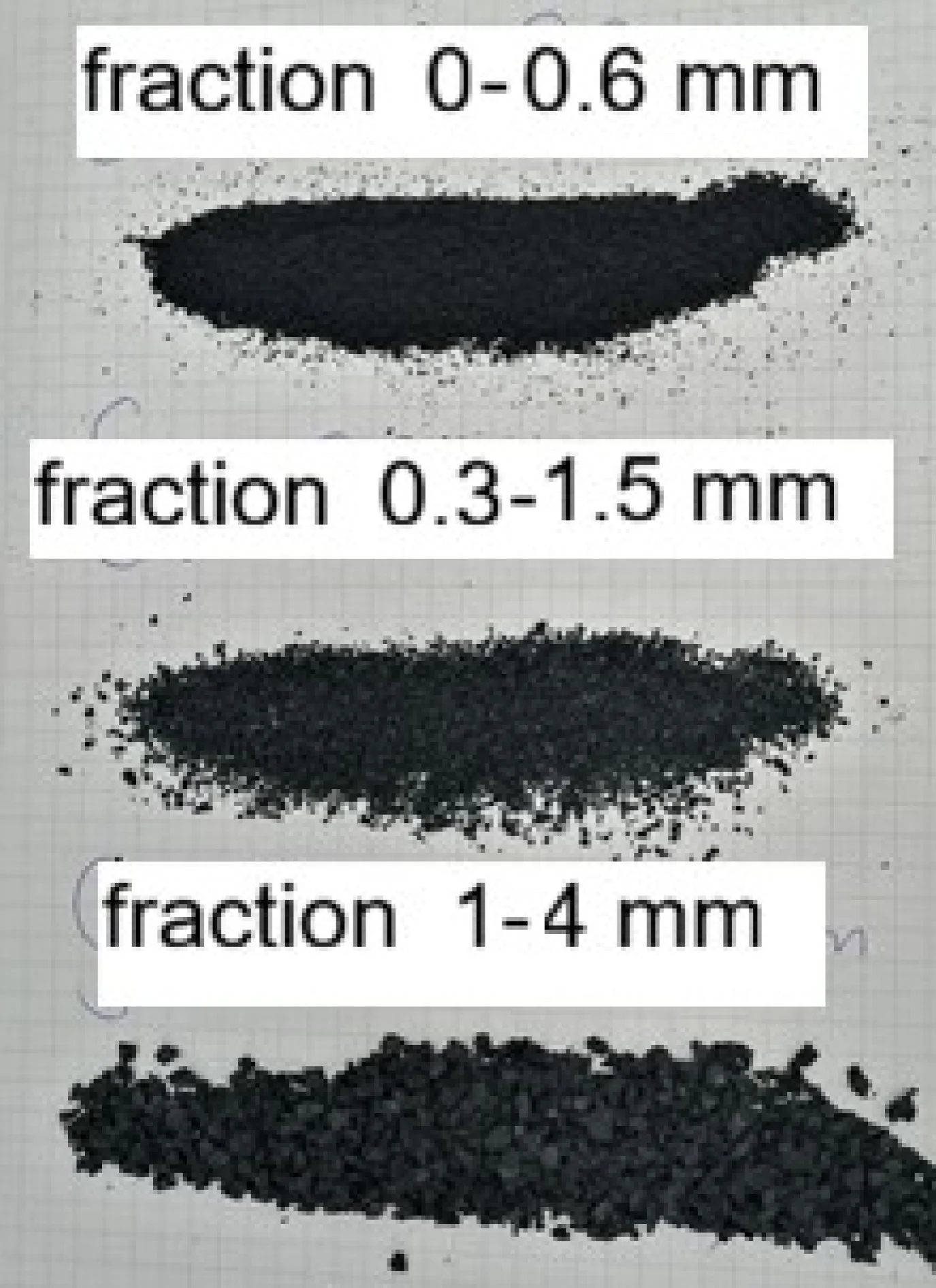
Rubber granulates used in the study.

As an additive to gypsum, rubber supplied by Recykl (Śrem, Poland) was used in the study in three different particle size fractions (Figure 1): rubber powder with a grain size range of 0–0.4 mm, and two rubber granulates with grain size ranges of 0.3–1.5 mm and 1–4 mm, respectively.

Rubber granulate is a material obtained through the mechanical shredding of tyres into particles with strictly controlled granulometry. The granulate is characterised by a bulk density in the range of 380–600 kg/m^3^ and a styrene–butadiene rubber (SBR) content of 60%, which ensures adequate elasticity and mechanical resistance of the material [30,31].

The rubber powder used in the study is a finely ground rubber material obtained by further shredding rubber granulate to particle sizes below 0.6 mm, typically within the range of 0.1–0.4 mm. The bulk density of the powder was approximately 400–600 kg/m^3^ [29].

### 2.3. Sample Preparation

In the case of gypsum pastes, commercially available gypsum (calcium sulfate hemihydrate) was mixed with distilled water. As shown in Table 2, gypsum was replaced with ground rubber at levels of 5%, 10%, 15%, 25%, and 50% by mass. All percentage values reported in the manuscript refer exclusively to mass replacement, with the amount of gypsum reduced accordingly while maintaining a constant water-to-gypsum ratio of 0.7.

In this study, a constant mass-based water-to-gypsum ratio of 0.70 was deliberately applied, referring exclusively to the mass of the active binder. This approach ensures consistent hydration conditions and enables a clear assessment of the influence of the mass replacement of gypsum with rubber on the mechanical properties of the composite. We acknowledge that substituting part of the gypsum with rubber changes the effective water-to-active-binder ratio, which may affect porosity and strength. However, the analysis of these effects was beyond the scope of the present work, whose objective was to evaluate the mechanical response of gypsum–rubber composites under fixed binding conditions. After combining the dry components with water, the mixtures were hand-mixed for 1 min and then poured into moulds.

### 2.4. Methods, Devices and Measurement Conditions

Compressive and flexural strength tests were carried out on gypsum–rubber composite samples formed into prismatic bars with nominal dimensions of 4 × 4 × 16 cm. For each mixture composition, six specimens were prepared. All specimens were first subjected to three-point flexural strength testing, with each bar yielding two fragments, resulting in twelve halves subsequently used for compressive strength testing. This procedure ensured full material consistency between flexural and compressive specimens and enabled the acquisition of comparable results within each measurement series. After casting, the samples were stored for 28 days at a temperature of 20 ± 1 °C and relative humidity of 50–55%, ensuring stable curing conditions. Flexural and compressive strength tests were performed using a modernised Hackert ZD10/90 testing machine (Fritz HECKERT, Chemnitz, Germany). The loading rate was set to 1 mm/min for flexural tests and 2 mm/min for compressive tests, in accordance with standard procedures applied for gypsum-based materials. A detailed description of the testing setup and procedures is provided in publications [35,36].

## 3. Results and Discussion

### 3.1. Density

Based on the dimensions and mass of the specimens, the density ρ values of all analysed composites were calculated. The results are summarised in Table 3.

The obtained density results show that an increase in the content of rubber granulate leads to a systematic reduction in the density of the gypsum composite across all analysed fractions. The highest density values were recorded for samples without the additive. However, even at a 5–10% content, a clear decrease in density is observed, particularly for the finest fraction of 0–0.6 mm. The differences between fractions are relatively small at low additive contents, but they become more pronounced at 50%, where the density of the samples decreases to 1031–1204 kg/m^3^. This effect indicates a strong loosening of the gypsum binder structure. The greatest density stability is maintained by gypsum containing the 1–4 mm rubber fraction, which suggests a more favourable incorporation of larger rubber particles into the binder structure. The results confirm that the rubber additive acts as a lightweight filler that significantly reduces the bulk density of the composite. At higher rubber contents (25–50%), the overall amount of the elastic phase becomes the dominant factor affecting density, which reduces the relative influence of particle size and makes the differences between fractions much less pronounced.

Based on the test results, a generalised relationship (1) was proposed, describing the density as a function of the mass fraction of rubber w_r_, expressed in percent, for all applied fractions:

(1) ρ = −A · w_r_ + B

The constants A and B of Equation (1), as well as the determination coefficients R^2^, are presented in Table 4.

### 3.2. Mechanical Strength Tests

**Figure 2:**
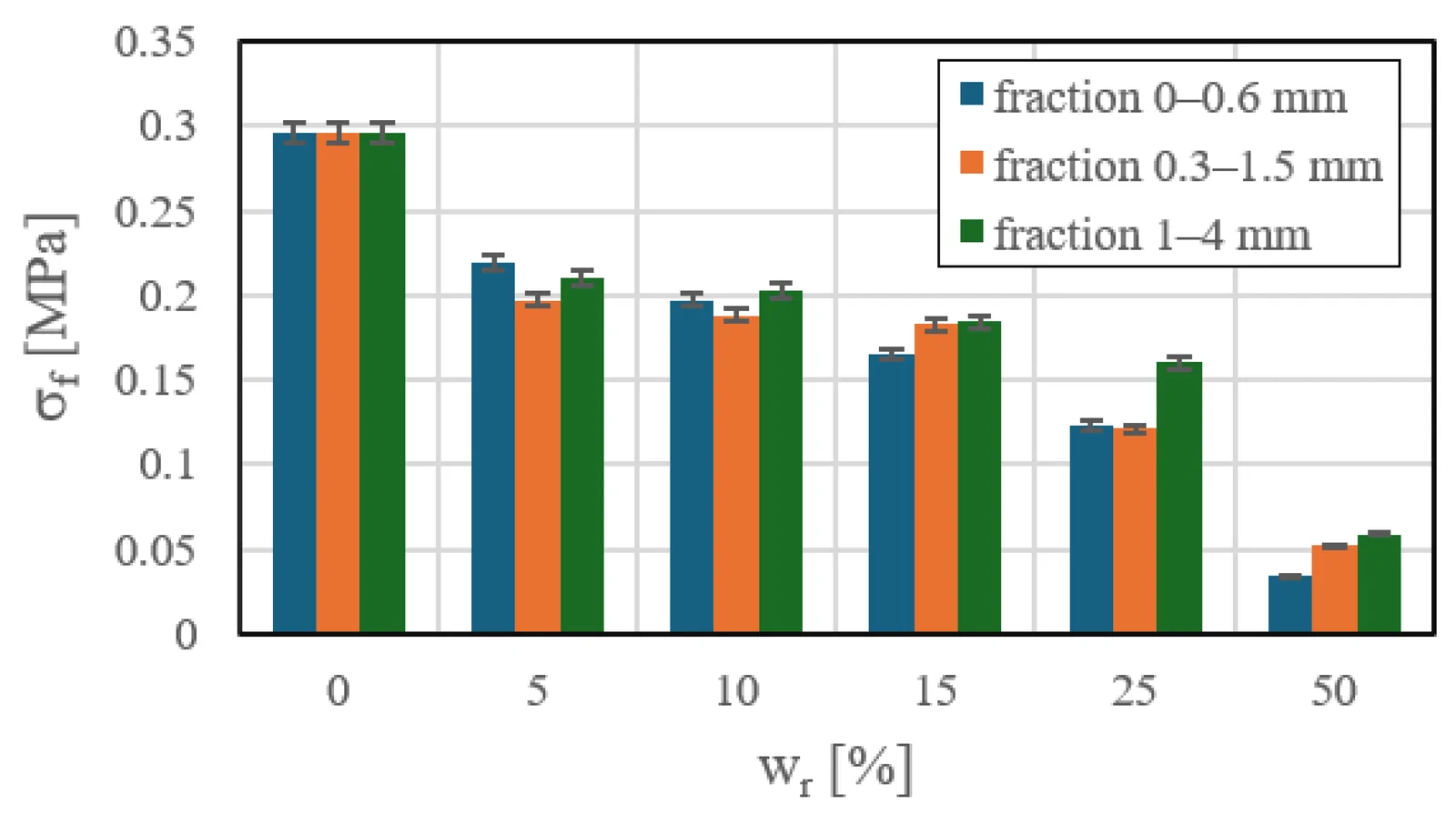
Influence of the rubber additive content on the flexural strength σ_f_ of the gypsum composite.

**Figure 3:**
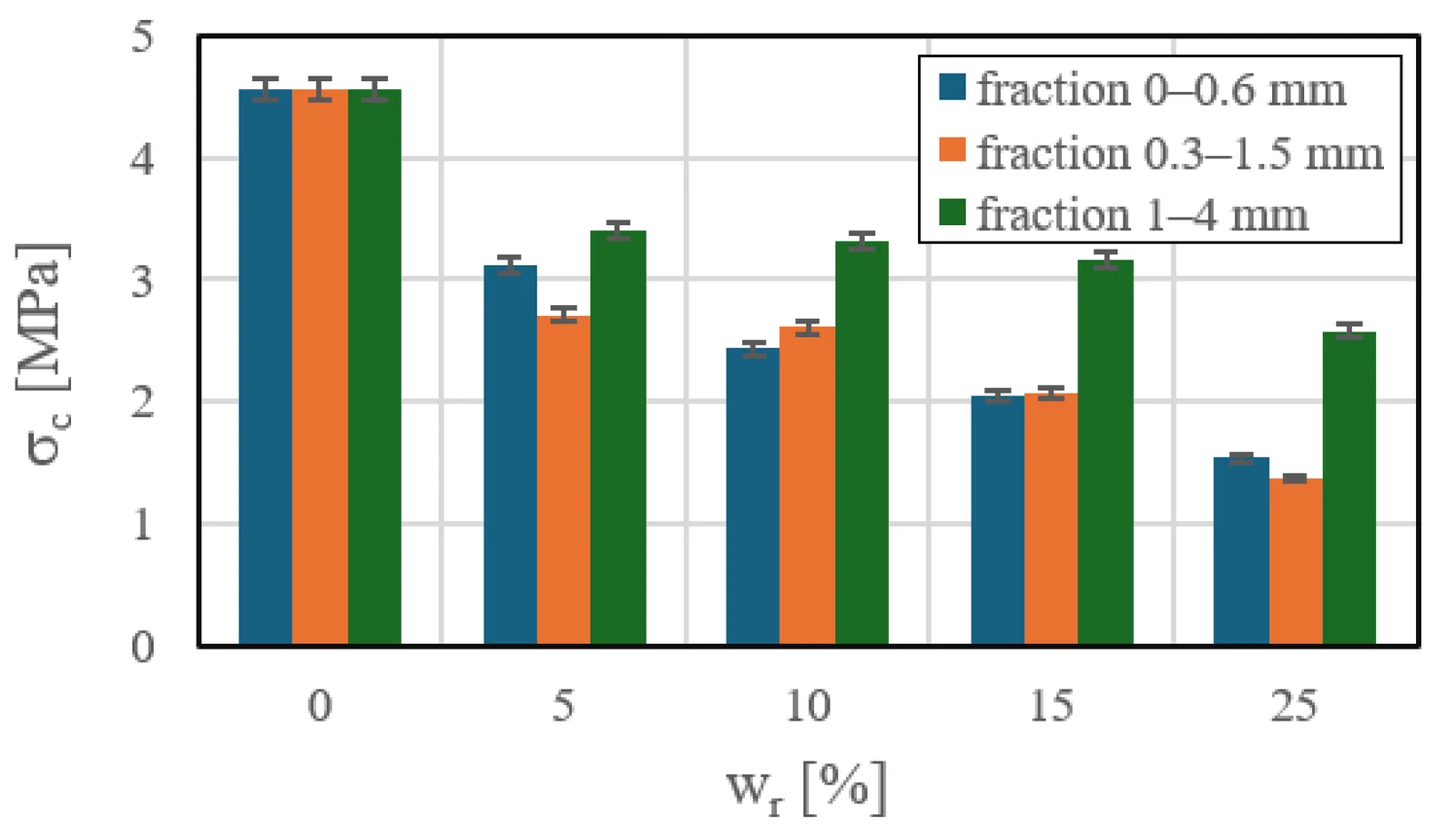
Influence of the rubber additive content on the compressive strength σ_c_ of the gypsum composite.

After 28 days of incubation and curing, the flexural strength tests σ_f_ and compressive strength tests σ_c_ were carried out as a function of the amount of rubber additive w_r_ and the grain size of the rubber particles. The obtained results are presented in Figure 2 and Figure 3. The testing procedure included three measurements of flexural strength and six measurements of compressive strength.

With increasing amounts of rubber added to the gypsum matrix, a systematic decrease in flexural strength was observed in all tested specimens, regardless of the particle-size fraction used. The highest flexural strength was recorded for pure gypsum, reaching 0.296 MPa. An additive content of 5–15% leads to a clear and gradual reduction in mechanical performance, while the differences between the fractions remain relatively small. The most pronounced reduction in strength occurs at additive contents of 25% and 50%, where the values drop to below 0.1–0.15 MPa. For the 5% additive level, gypsum containing the 0–0.6 mm fraction exhibits slightly higher strength values compared with the other two fractions. The differences between the fractions become more noticeable as the additive content increases, suggesting a growing influence of the rubber particles on the structural integrity of the composite. The overall trend indicates that increasing the proportion of the additive weakens the material regardless of the particle-size fraction used. This behaviour may result from the disruption of continuity within the gypsum binder and the reduced efficiency of stress transfer.

The presented results clearly show that increasing the amount of rubber additive leads to a systematic decrease in compressive strength. This phenomenon is observed in all three analysed composites containing different particle-size fractions, although its intensity varies between them. The highest compressive strength was recorded for the reference sample without ground rubber, reaching 4.556 MPa.

For the modified samples, the highest compressive strength values were obtained for the 1–4 mm fraction, which suggests a more favourable interaction between the larger particles and the gypsum matrix, whereas the fractions with smaller particle diameters exhibit lower strength values, likely due to greater structural disruption and a weaker filling effect within the composite. As the additive content increases to 25%, the reduction in strength becomes particularly pronounced, indicating that the optimal amount of the modifying material has been exceeded, and the differences between the fractions are then most evident. At low additive contents, the decreases in strength are relatively mild, suggesting that a small amount of the additive does not significantly disturb the structure of the composite.

An excessive amount of the additive leads to the formation of voids and discontinuities within the material structure, resulting in a decrease in its strength. The sample containing 50% rubber granulate was not suitable for compressive strength testing, as it had completely disintegrated during the earlier flexural strength tests, which made its further use in the testing procedure impossible. The pronounced reduction in mechanical strength observed at higher rubber contents can be attributed to several interacting mechanisms. Increasing the proportion of rubber decreases the volume of the active gypsum matrix, which directly limits the load-bearing capacity of the composite. At the same time, the incorporation of elastic particles disrupts the continuity of the binder and increases overall porosity, particularly in mixtures containing fine rubber fractions. The gypsum–rubber interface forms a transition zone with limited adhesion, which promotes local stress concentrations and facilitates the initiation of microcracks under load. Additionally, the packing of rubber particles becomes less efficient at higher replacement levels, leading to the formation of voids and discontinuities that further weaken the structure. The inherently elastic nature of rubber also reduces the stiffness of the composite, diminishing its ability to transfer stresses effectively. These combined effects explain the significant decrease in both flexural and compressive strength at high rubber contents.

The differences between fractions indicate that not only the amount of the additive but also its granulation plays a key role in determining mechanical properties, with the largest fraction proving to be the most resistant to the negative effects of increasing additive content. The results suggest that an appropriate selection of particle-size fraction may partially compensate for the strength reduction caused by higher additive levels, and the clear decreasing trend observed for each fraction confirms the predictability of the material’s behaviour and may be used to model composite properties as a function of component proportions. The data also indicate that, when designing the mixtures, particular attention should be paid to the share of fine fractions, as their excess may lead to a significant deterioration of performance parameters, whereas coarser fractions may offer a favourable compromise between structural modification and maintaining strength. Larger rubber particles interact less effectively with the gypsum matrix, creating more pronounced interfacial transition zones and local voids, which reduces stress-transfer efficiency and leads to less uniform particle distribution within the composite. In the investigated range, rubber acts primarily as a lightweight elastic filler that reduces the active binder volume, and therefore does not improve mechanical strength parameters compared with pure gypsum.

These observations are consistent with trends reported in studies on rubber-modified gypsum and cement composites, where increasing rubber content typically reduces strength due to higher porosity and limited binder continuity [37,38,39].

Based on the test results, generalised relationships (2) and (3) were proposed for all examined samples, describing the flexural strength σ_f1_ as a function of density and the compressive strength σ_c1_ as a function of density:

(2) σ_f1_ = 0.0005 · ρ − 0.4988

(3) σ_c1_ = 0.0110 · ρ − 13.046

It was also possible to propose generalised relationships (4) and (5), describing the flexural strength σ_f2_ and the compressive strength σ_c2_ as a function of the rubber mass fraction w_r_, expressed in percent:

(4) σ_f2_ = −0.0043 · w_r_ + 0.2529

(5) σ_c2_ = −0.0975 · w_r_ + 4.0077

**Figure 4:**
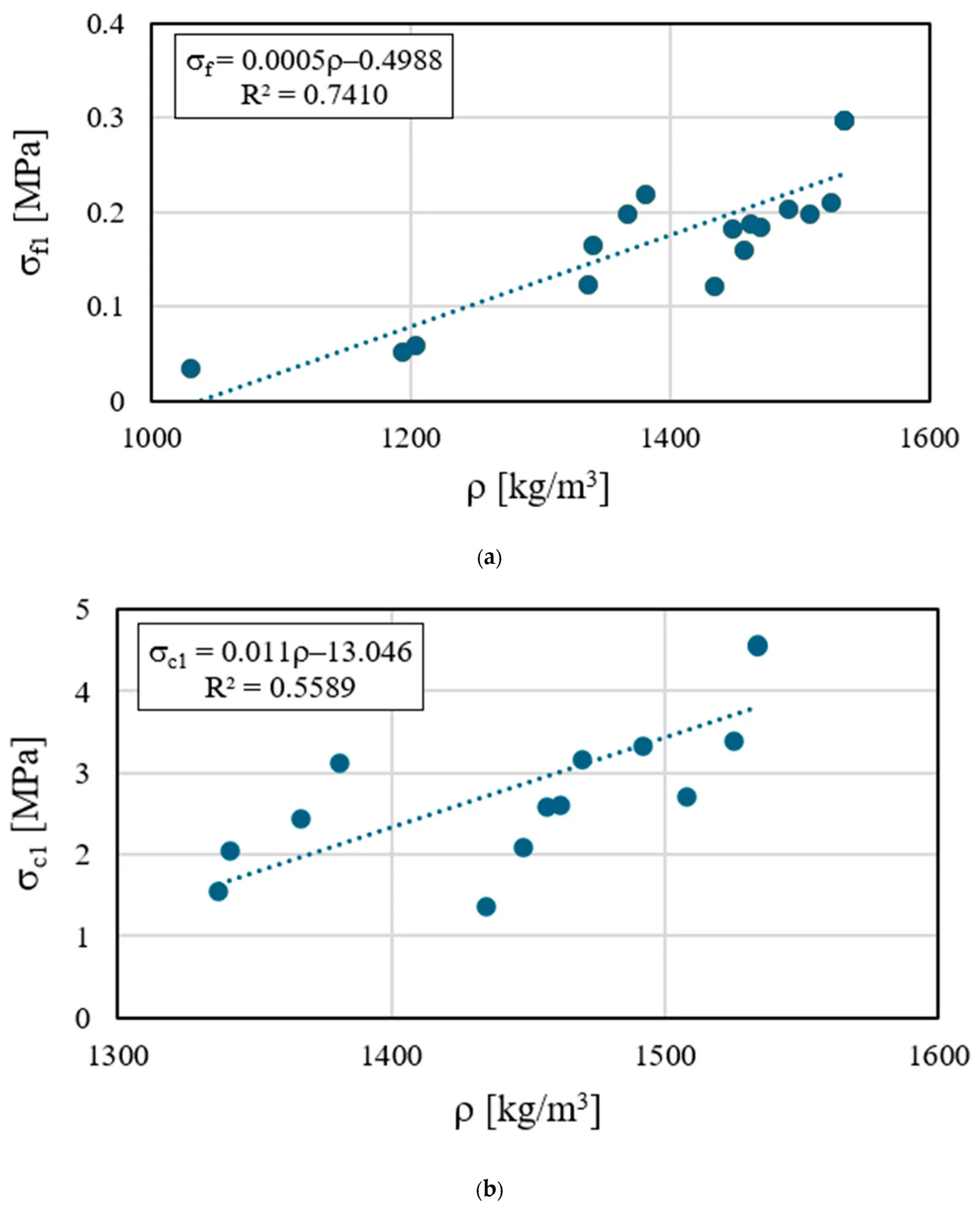
Graph of the relationship between flexural strength σ_f1_ (**a**) and compressive strength σ_c1_ (**b**) as a function of sample density ρ used in the study.

Figure 4a,b present the generalised plots of the relationships σ_f1_ = f(ρ) (Figure 4a) and σ_c1_ = f(ρ) (Figure 4b) for all tested samples.

**Figure 5:**
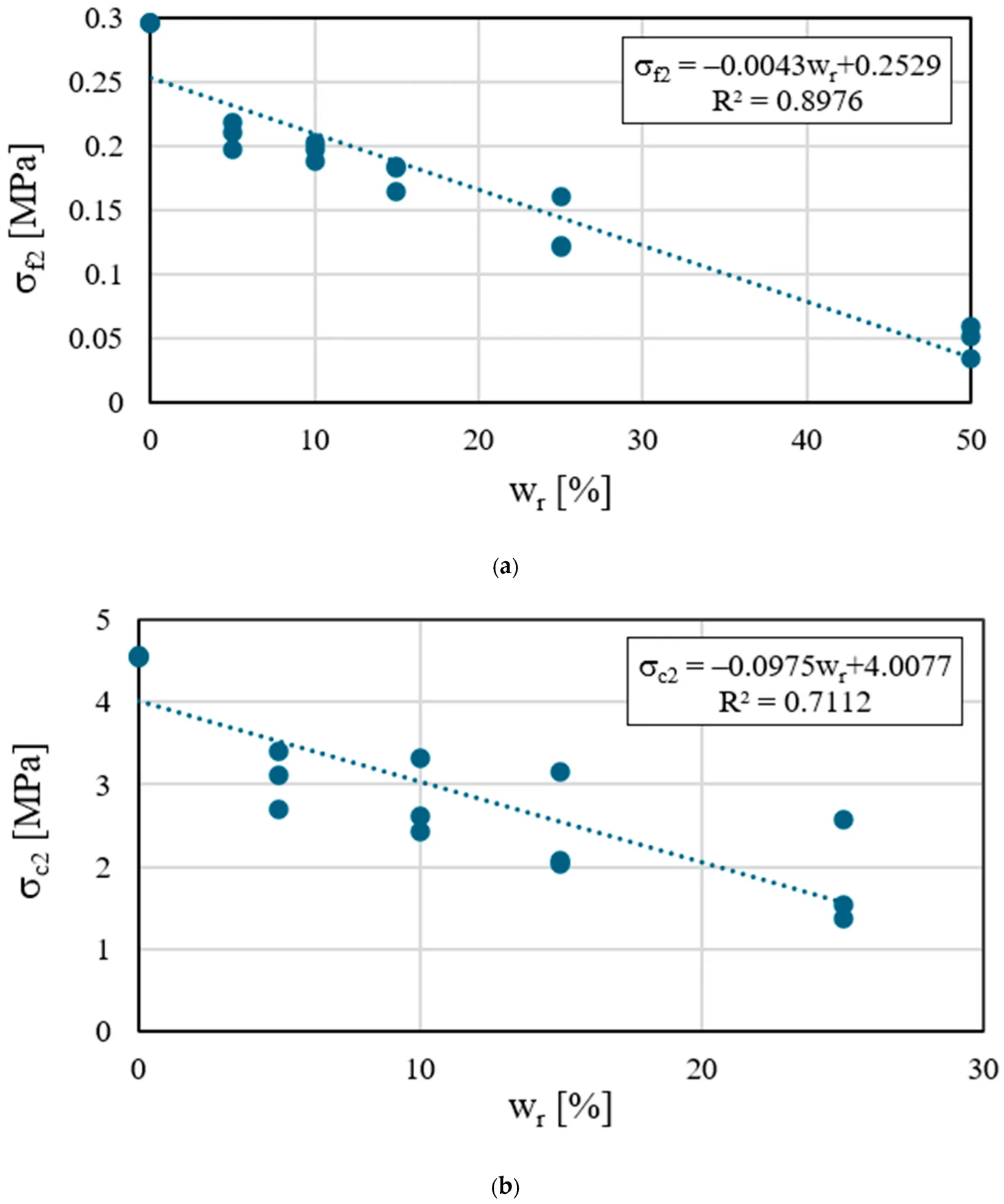
Graph of the relationship between flexural strength σ_f2_ (**a**) and compressive strength σ_c2_ (**b**) as a function of the rubber mass fraction w_r_ in the samples used in the study.

Analogously, Figure 5a,b present the generalised graphs of the relationships σ_f2_ = f(w_r_) (Figure 5a) and σ_c2_ = f(w_r_) (Figure 5b) for all tested samples.

The relatively low R^2^ values result from the multiscale nature of the gypsum–rubber composite. Changes in rubber content simultaneously affect porosity, binder continuity, interfacial transition zones and particle distribution, which cannot be fully captured by a single-parameter linear model. Therefore, the proposed correlations should be interpreted as descriptive tools for trend estimation rather than precise predictive models. The proposed Equations (2)–(5) allow for a fast and, at the same time, accurate calculation of the flexural strength σf and the compressive strength σc of the samples, based solely on their density or the amount of rubber used. Such estimation does not require performing destructive tests on the available samples.

Based on the test results, the basic statistical parameters of the experimental values of σf and σc were determined and compiled in Table 5 and Table 6. This enabled a quick comparison of the values obtained in the individual series. The high porosity and brittleness of the samples with a 50% addition of rubber granulate meant that, after completing the flexural strength tests, they were no longer suitable for further compressive strength testing.

## 4. Model Validation—Analysis of Two Regression Approaches

**Figure 6:**
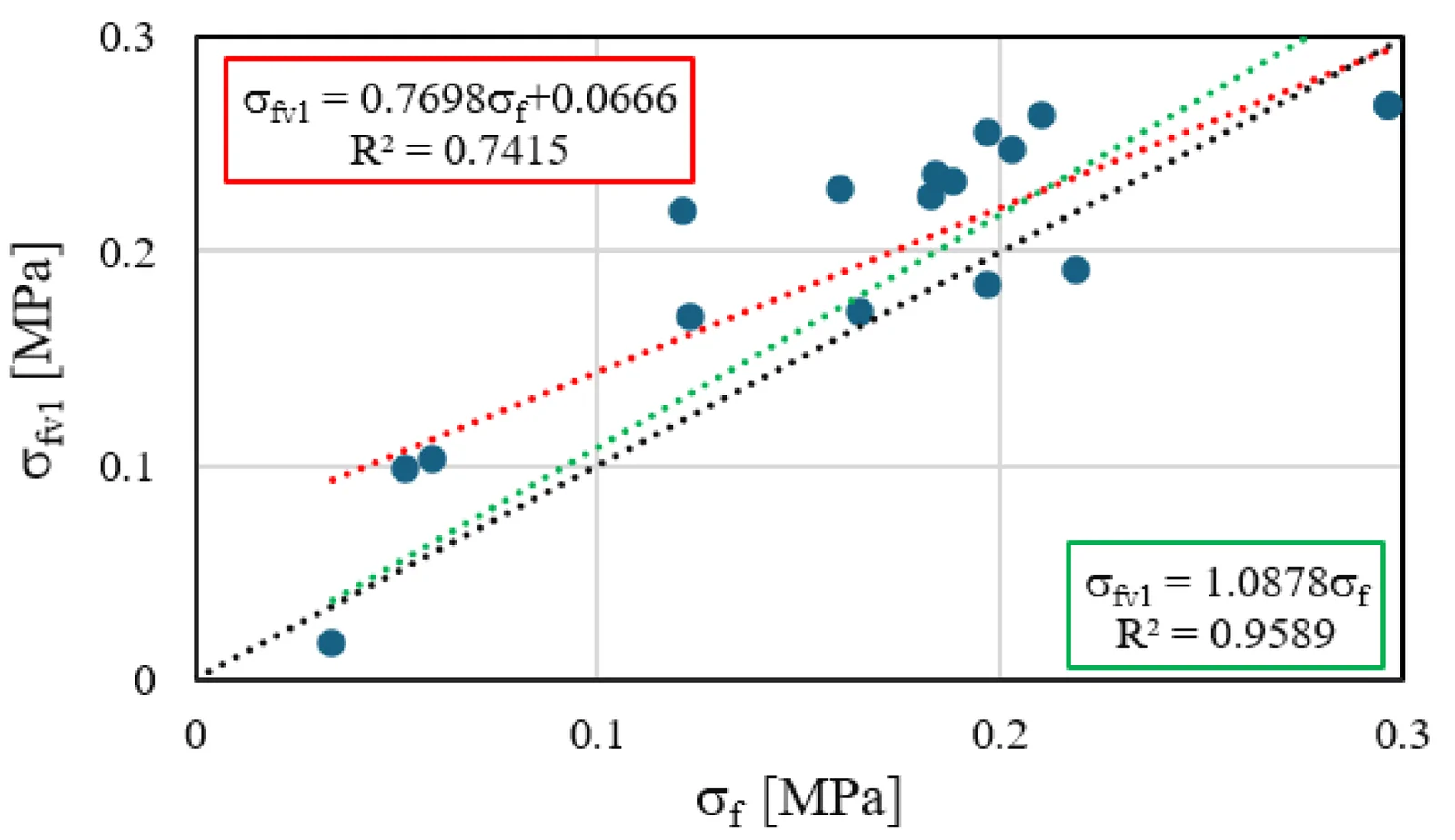
Comparison of the flexural strength σ_f_ values obtained experimentally and the calculated σ_fv1_ values based on Equation (2), including linear regression with an intercept and regression forced through the origin (0, 0).

To assess the agreement between the calculated and experimental values, a comparative analysis was carried out for the flexural strength σf and the corresponding validation values σ_fv1_ obtained from Equation (2). This relationship is presented in Figure 6, which illustrates two complementary perspectives on the quality of the fit.

The first approach (red line) involves linear regression with an intercept. This formulation reflects the actual distribution of the data points and makes it possible to assess whether systematic deviations occur between the experimental and calculated values. The resulting relationship, σ_fv1_ = 0.7698σ_f_ + 0.0666 (R^2^ = 0.7415), indicates a slight underestimation of higher values and a minor overestimation of the lowest ones. This is a natural consequence of the scatter inherent in the measurement data and provides diagnostic insight into the nature of the model’s errors. The second approach (green line) forces the regression line to pass through the origin (0, 0), which corresponds to the physical assumption that the calculated values should equal the experimental ones for any rubber content. In this case, the formulation serves exclusively to evaluate the proportionality between the experimental and calculated values. The obtained relationship, σ_fv1_ = 1.0878σ_f_ (R^2^ = 0.9589), demonstrates high linearity under the proportionality constraint while also revealing a slight tendency toward overestimation. Both approaches are complementary: the free regression assesses the quality of the fit and identifies systematic errors, whereas the forced regression verifies whether the relationship exhibits proportional behaviour relative to the ideal line y = x (black line).

**Figure 7:**
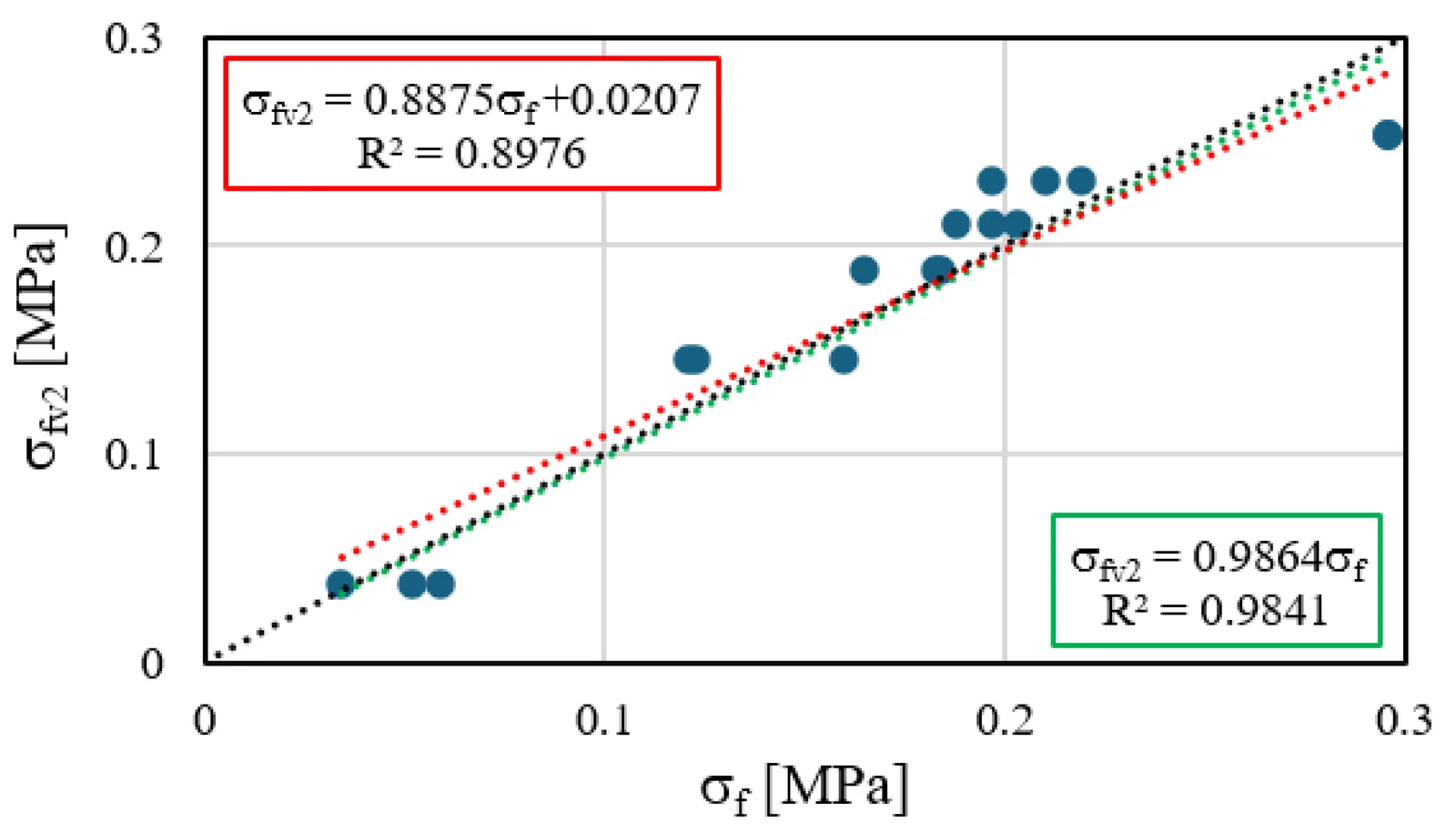
Comparison of the flexural strength σ_f_ values obtained experimentally and the calculated σ_fv2_ values based on Equation (4), including linear regression with an intercept and regression forced through the origin (0, 0).

An analogous validation was performed for the proposed correlation (4), taking into account the amount of rubber mixture used in the calculations. Figure 7 presents two approaches, encompassing linear regression with an intercept as well as regression forced to pass through the origin (0, 0).

**Figure 8:**
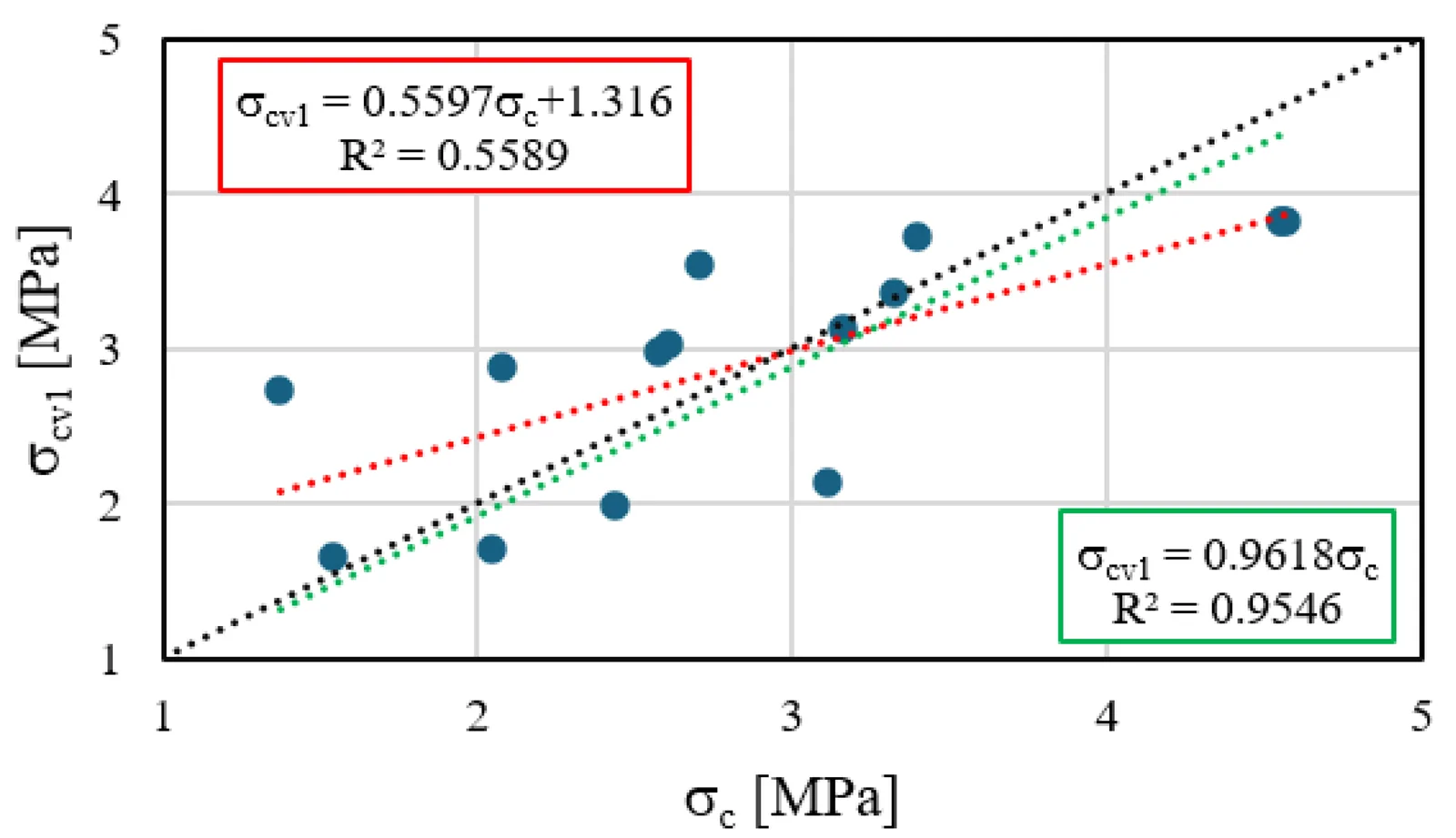
Comparison of the compressive strength σ_c_ values obtained experimentally and the calculated σ_cv1_ values based on Equation (3), including linear regression with an intercept and regression forced through the origin (0, 0).

**Figure 9:**
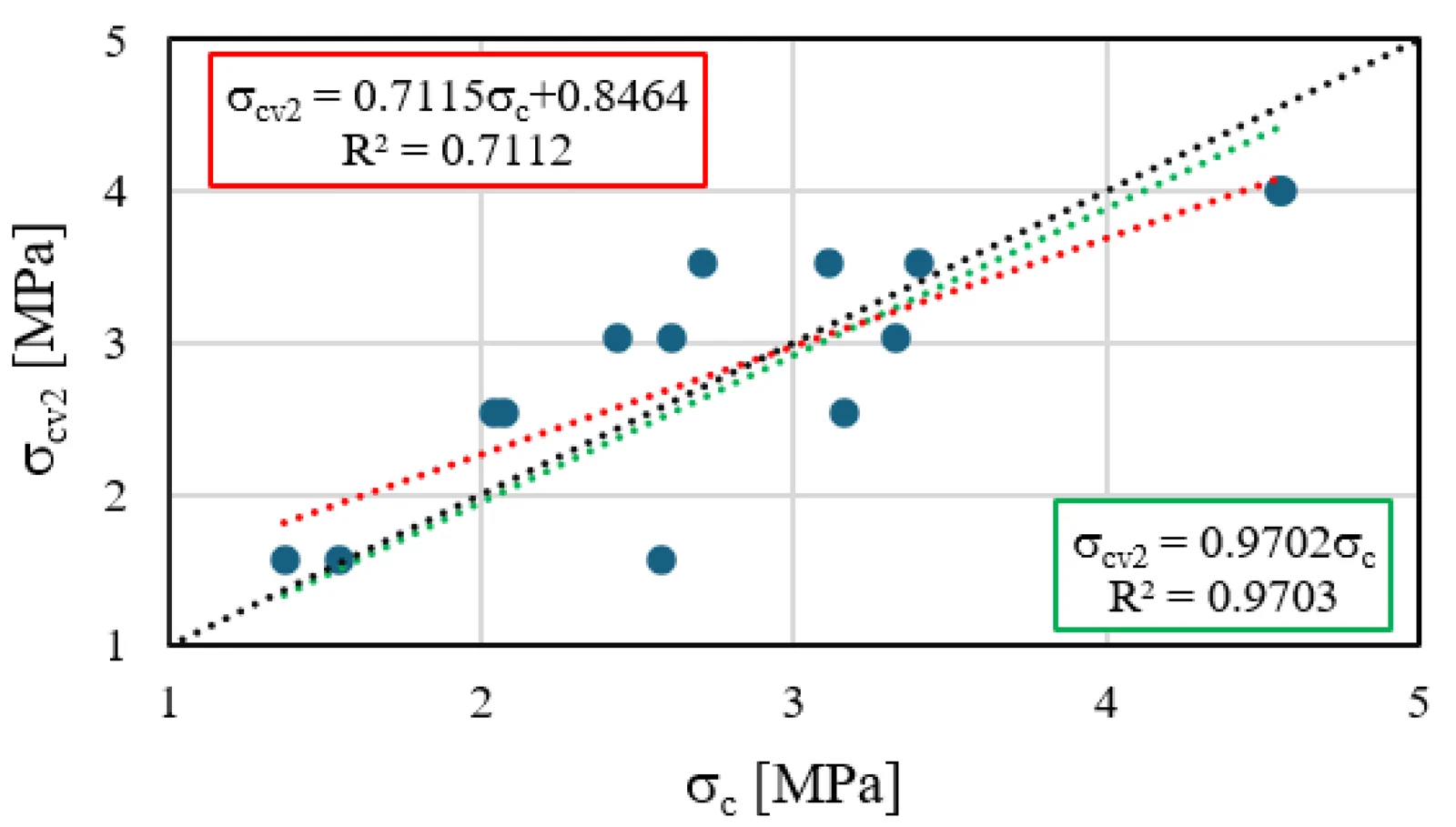
Comparison of the compressive strength σ_c_ values obtained experimentally and the calculated σ_cv2_ values based on Equation (5), including linear regression with an intercept and regression forced through the origin (0, 0).

A corresponding validation was carried out for the proposed correlations (3) and (5), related to the experimental determination of the compressive strength σ_c_ and the calculated values σ_cv1_ and σ_cv2_. The results are presented in Figure 8 and Figure 9.

The graphs presented in Figure 6, Figure 7, Figure 8 and Figure 9 enable a quantitative assessment of how well the regression models agree with the experimental data by analysing two key parameters: the slope *a* and the coefficient of determination *R*^2^. In the case of free regression (with an intercept), slope values *a* deviating from 1 and a positive intercept *b* indicate a systematic shift in the regression line relative to the measurement data. It was observed that in all cases the slope *a* is lower than 1, meaning that the proposed equations respond more weakly to increases in the experimental values, leading to an underestimation of higher values and an overestimation of lower ones. This behaviour is a typical indication that the error variance increases with the magnitude of the independent variable.

The regressions forced through the origin allow the assessment of the purely proportional character of the relationships. High values of R^2^ (above 0.95) in all analysed cases indicate that the variability of the data is largely explained by the linear relationship, and that the deviations of the points from the regression line are predominantly random. The slope values *a* differ between the individual relationships: in three cases *a* is lower than 1, which indicates a tendency to underestimate the calculated values relative to the experimental ones, whereas in one case *a* = 1.0878, pointing to a slight tendency toward overestimation. From a mathematical perspective, this means that the sensitivity of the linear relationship to changes in the input (experimental) values may be slightly lower or slightly higher than in the real system, depending on the property being analysed.

The comparison of the graphs for flexural and compressive strength reveals clear differences in the character of data scatter. In the case of compressive strength, the measurement points cluster more closely around the regression line, resulting in higher values of the coefficient of determination and lower variability of the model error. The proposed equations therefore reproduce the linear relationship between the analysed quantities more accurately. A different pattern is observed for flexural strength, where the greater scatter of points indicates higher material heterogeneity and a stronger sensitivity of this property to local defects. Statistically, this translates into greater error variability and lower stability of the slope parameter.

It is also worth noting the nature of the deviations of the points from the regression line. In free regressions, the deviations exhibit slight asymmetry, suggesting that the model does not fit equally well across the entire range of analysed values. In forced regressions, the deviations are more symmetrical, although their absolute magnitude increases at higher values of the independent variable, which is a typical phenomenon observed in material testing.

Overall, the graphs confirm that the proposed models exhibit a linear character and that their fit is satisfactory in both the free and forced approaches. Free regression provides information on systematic deviations of the model, whereas forced regression allows the assessment of proportionality and the stability of the slope parameter. Both approaches are complementary and together enable a comprehensive evaluation of the quality of the correlations.

To evaluate the quality of the fit between the computational models and the experimental data, three standard error metrics were employed: the Sum of Squared Errors (SSE), the Root Mean Square Error (RMS), and the Mean Absolute Percentage Error (MAPE). Each of these measures captures a different aspect of the agreement between the calculated values and the actual measurement results.

SSE is defined as the sum of the squared differences between the experimental values *y_i_* and the calculated values ${\hat{y}}_{i}$ (6):

(6) $$SSE=\sum_{i=1}^{n}(y_{i}-{\hat{y}}_{i}{)}^{2}$$

This metric reflects the global level of model error—the lower the SSE value, the better the fit. High values indicate the presence of substantial local deviations.

The RMS is the square root of the mean squared error (7):

(7) $$RMS=\sqrt{\frac{1}{n}\sum_{i=1}^{n}(y_{i}-{\hat{y}}_{i}{)}^{2}}$$

The RMS expresses the average magnitude of the model error, expressed in the units of the analysed property. This metric is particularly sensitive to large deviations because the errors are squared.

The third metric is the MAPE, which describes the relative error of the model (8):

(8) $$MAPE=\frac{1}{n}\sum_{i=1}^{n}|\frac{y_{i}-{\hat{y}}_{i}}{y_{i}}|$$

The MAPE makes it possible to assess how large the model error is relative to the experimental values, which is particularly important when the data span different scales.

The analysis of the RMS, MAPE, and SSE values (Table 7) clearly indicates that the equations describing compressive strength exhibit substantially better fit than those describing flexural strength. This results from the lower variability of the data and the more distinct, well-defined character of the relationship in the case of compression.

In the case of bending, the model described by Equation (2) exhibits higher RMS and SSE values than the one described by Equation (4), which indicates a greater dispersion of residuals and the presence of local deviations. The model based on Equation (4) is characterised by lower values of all three metrics, confirming its more stable and proportional representation of the experimental data. The lower MAPE value for Equation (4) indicates that the relative error of the model is smaller, and therefore it preserves the relationships between the values more accurately.

In the case of compression, the RMS and SSE values (Equations (3) and (5)) are higher than those obtained for bending (Equations (2) and (4)), which results from the greater absolute variability of the experimental data for this property. Nevertheless, both models maintain good agreement with the measurements, as indicated by the relatively low MAPE values. Model (5) stands out with the lowest MAPE among all analysed results, which means that it most accurately reflects the relative changes in compressive strength. Model (3) exhibits higher SSE, which stems from its tendency to systematically underestimate values at higher load levels.

In summary, the highest quality of fit among all analysed equations was obtained for model (5) in the compression domain, whereas in the case of bending, model (4) demonstrated the best fit, as confirmed by all evaluated error metrics.

## 5. Conclusions

Understanding the influence of rubber derived from the vulcanisation industry on the properties of gypsum composites has both scientific and practical significance. It enables the prediction of material behaviour, the design of composites with defined parameters, and the assessment of the potential for using rubber waste in construction. Based on the conducted research, the following conclusions were drawn.

### 5.1. Experimental Findings

1. The addition of rubber granulate leads to a systematic decrease in the density of gypsum composites across all analysed fractions. The strongest reduction occurs at high additive contents (25–50%). Among the tested fractions, the 1–4 mm granulate maintains the highest density stability.
2. Flexural and compressive strength decrease with increasing rubber content. The highest strength values were obtained for the reference samples, whereas the largest reductions were observed at additive contents of 25% and 50%. The 1–4 mm fraction consistently produced higher strength values compared with the finer fractions.
3. The proposed regression equations accurately describe the relationships between flexural/compressive strength and density, as well as between flexural/compressive strength and rubber mass content. Both free and forced linear regression variants exhibit high agreement with the experimental data.
4. Validation error analysis (RMS, MAPE, SSE) shows that the models describing compressive strength achieve a better fit than those describing flexural strength.

### 5.2. Interpreted Mechanisms

1. The observed decrease in density results from the lower density of rubber compared with gypsum and from the loosening of the binder structure caused by the introduction of elastic particles.
2. The reduction in mechanical strength is attributed to the disruption of structural continuity and the limited efficiency of stress transfer within the composite. Larger rubber particles (1–4 mm) appear to integrate more favourably with the gypsum matrix, which may explain their comparatively higher strength values.
3. The regression models provide a practical tool for estimating mechanical properties without destructive testing, supporting the design of composites with tailored performance.
4. The incorporation of rubber granulate represents a promising approach to modifying gypsum-based materials within the circular-economy framework. Although the addition of rubber reduces strength, it improves deformation resistance and enables the production of lighter composites. Appropriate selection of fraction size and additive content allows partial compensation of these effects and supports the development of materials with balanced properties.

## Figures and Tables

**Table 1:** Chemical formulas of the primary rubbers used in tyre manufacturing.

Name of Rubber	Monomers	Polymer Formula
Natural Rubber (NR)	CH_2_=C(CH_3_)–CH=CH_2_	[–CH_2_–C(CH_3_)=CH–CH_2_–]_n_
Butadiene Rubber (BR)	CH_2_=CH–CH=CH_2_	[–CH_2_–CH=CH–CH_2_–]_n_
Styrene Butadiene Rubber (SBR)	CH_2_=CH–CH=CH_2_ C_6_H_5_–CH=CH_2_	[–CH_2_–CH=CH–CH_2_–]_x_– [–CH_2_–CH(C_6_H_5_)–]_y_
Isobutylene Isoprene Rubber (IIR)	CH_2_=C(CH_3_)_2_ CH_2_=C(CH_3_)–CH=CH_2_	[–CH_2_–C(CH_3_)_2_–]_x_– [–CH_2_–C(CH_3_)=CH–CH_2_–]_y_

**Table 2:** Components of the gypsum composites used in the study.

Additive Content [%]	Gypsum [g]	Water [g]	Ground Rubber [g]
0	1750	1225	0
5	1662.5	1163.8	87.5
10	1675	1102.5	175
15	1487.5	1041.3	262.5
25	1312.5	918.8	437.5
50	875	612.5	875

**Table 3:** Calculated density ρ values of the specimens used in the study.

Additive Content [%]	Density ρ [kg/m^3^]		
	Fraction 0–0.6 [mm]	Fraction 0.3–1.5 [mm]	Fraction 1–4 [mm]
0	1534	1534	1534
5	1381	1508	1525
10	1367	1462	1492
15	1341	1448	1470
25	1337	1435	1457
50	1031	1194	1204

**Table 4:** Constants A and B and the determination coefficient R^2^ of Equation (1).

Fraction [mm]	A	B	R^2^
0–0.6	8.658	1483.3	0.9083
0.3–1.5	6.542	1544.7	0.9474
1–4	6.544	1561.5	0.9304

**Table 5:** Basic statistical parameters determined from the flexural strength σ_f_ tests of the modified gypsum samples.

Additive Content [%]	Flexural Strength σ_f_		
	Mean Value [MPa]	Standard Deviation [MPa]	Coefficient of Variation [%]
0	0.296	0.019	6.3
	Fraction 0–0.6 [mm]		
5	0.219	0.023	10.7
10	0.197	0.043	21.9
15	0.147	0.031	20.9
25	0.123	0.004	2.9
50	0.033	0.003	7.6
	Fraction 0.3–1.5 [mm]		
5	0.197	0.038	19.3
10	0.188	0.012	6.6
15	0.183	0.021	11.5
25	0.121	0.033	27.7
50	0.051	0.009	16.6
	Fraction 1–4 [mm]		
5	0.210	0.048	22.7
10	0.203	0.022	10.9
15	0.184	0.058	31.7
25	0.160	0.053	33.2
50	0.059	0.021	34.7

**Table 6:** Basic statistical parameters determined from the compressive strength σ_c_ tests of the modified gypsum samples.

Additive Content [%]	Compressive Strength σ_c_		
	Mean Value [MPa]	Standard Deviation [MPa]	Coefficient of Variation [%]
0	4.566	0.323	7.1
	Fraction 0–0.6 [mm]		
5	3.115	0.540	17.3
10	2.453	0.300	12.2
15	2.042	0.098	4.8
25	1.542	0.133	8.7
	Fraction 0.3–1.5 [mm]		
5	2.709	0.136	5.0
10	2.609	0.088	3.4
15	2.076	0.063	3.0
25	1.370	0.121	8.9
	Fraction 1–4 [mm]		
5	4.566	0.323	7.1
10	3.326	0.321	9.7
15	3.159	0.468	14.8
25	2.578	0.175	6.8

**Table 7:** The calculated values of the error metrics (SSE, RMS, and MAPE).

Measured Property	Equation	SSE [MPa^2^]	RMS [MPa]	MAPE [-]
Flexural strength σ_f_	(2)	0.039	0.047	0.322
Flexural strength σ_f_	(4)	0.017	0.031	0.188
Compressive strength σ_c_	(3)	2.319	0.393	0.128
Compressive strength σ_c_	(5)	0.643	0.207	0.067

## Data Availability

The original contributions presented in this study are included in the article. Further inquiries can be directed to the corresponding author.
